# Public Mental Health – Using the Mental Health Gap Action Program to Put all Hands to the Pumps

**DOI:** 10.3389/fpubh.2014.00033

**Published:** 2014-04-22

**Authors:** Richard Uwakwe, Alex Otakpor

**Affiliations:** ^1^Faculty of Medicine, Nnamdi Azikiwe University, Awka, Nigeria; ^2^Department of Mental Health, University of Benin, Benin City, Nigeria

**Keywords:** pathway to care, religious and healers, biomedical medicine, collaboration, developing countries

## Abstract

Although mental ill health constitutes a huge portion of the Global Burden of Disease (GBD), the majority of people with mental health problems do not receive any treatment, a scenario much worse in developing countries where mental health personnel are in gross short supply. The mhGAP was launched to address this gap, especially by training non-mental health professionals to deliver effective services for selected priority mental health problems. Especially in developing countries, many people with mental health problems consult traditional healers either as a first step in the pathway to biomedical mental health care or as the sole mental health service providers. Bridging the gap between mental health needs and available services in developing countries needs to incorporate traditional healers, who are ubiquitously available, easily accessible, and acceptable to the natives. Even though there are barriers in forging collaborations between traditional and biomedical mental health care providers, with mutual respect, understanding, and adapted training using the mhGAP intervention guide, it should be possible to get some traditional healers to understand the core principles of some priority mental health problems identification, treatment, and referral.

## Background

Refinements in the tools and methodologies of psychiatric epidemiology have raised our understanding of the burden of mental disorders. More than a decade ago, the WHO reported that at any point in time no less than 10% of the population has mental disorders and at some point in their life 25% will develop mental disorders (1).

In a more recent report of the World Mental Health (WMH) Survey, it was reported that the prevalence of any WMH-CIDI/DSM-IV disorder in the prior year varied, from 4.3% in Shanghai to 26.4% in the United States. A good proportion of the 12-month cases were mild (between 33.1% in Colombia and 80.9% in Nigeria). The WMH authors concluded that serious (mental) disorders were associated with substantial role disability (2).

Moving away from the usual measure of only mortality, the collaboration between the WHO, World Bank, and Harvard University on the Global Burden of Disease (GBD) Project utilized the disability adjusted life year (DALY) – a summation of the years of life lost to premature mortality and years lost to disability (YLL + YLD). It was shown that neuropsychiatric conditions are responsible for 14% of the GBD, with unipolar depression ranking as the fourth most burdensome condition (3, 4).

Considering mortality, it has been estimated that one million suicides occur annually (5), with about 90% of the cases being associated with depression. According to WHO, this roughly translates to one suicide death every 40 s. Suicide ranks among the three leading causes of mortality among 15–45 years olds (for both genders). There is also reduced life expectancy in persons with schizophrenia, learning disabilities, dementia, bipolar disorders, and other major mental disorders.

In 2007, the Lancet published a series on global mental health (6). These series, *inter alia*, contain three core observations:

1. Mental disorders represent a substantial “though largely hidden” proportion of the world’s overall disease burden.
2. Every year up to 30% of the global population will develop some sort of mental disorder.
3. There is strong evidence for scaling up mental health services worldwide.
  To what extent are these burdens being addressed by the present system of health care delivery? According to the WMH Survey, the majority of those who need mental health care globally do not receive any treatment. In developed and developing countries, respectively, 35.5–50.3 and 76.3–85.4% of people with mental disorders had not received any form of treatment in the previous 12 months (2).
  Low and middle income countries (LAMIC) have very low levels of personnel and resources for mental health services. For example, the WHO Mental Health Atlas reports the following staff to population ratios per 100,000 populations for Nigeria: 0.06 psychiatrists, 0.02 psychologists, 0.19 nurses, 0.09 non-specialized doctors (7). Especially in LAMIC, mental health resources are also unevenly spread between urban/rural and north/south locations, etc.
  Thus there are huge gaps between needs and available services both within and between countries.

The Mental Health Gap Action Program (mhGAP) was officially launched by the WHO to address this gap in treatment for individuals with mental, neurological, and substance use disorders (8). One of the principal keys to success in the mhGAP program is partnerships. The WHO maintains that countries are to make the decisions as to how best to deliver the mhGAP intervention packages at health facility, community, and household levels to ensure high quality and equitable coverage. In other words, the major task is to identify the people who will be responsible for the delivery of the mhGAP interventions at each level of service delivery. To identify those who will deliver the mhGAP services at any level, it is necessary to know where to locate those who have mental, neurological, and substance disorders.

## Who Offers Mental Health Services Across the World?

All over the world, people living with mental, neurological, and substance disorders, when they do seek treatment, do so in a wide variety of sources, both formal and informal.

In describing the pathways to mental health care in eight countries in Eastern Europe, Gater et al. (9) reported that between 6 and 10% of the participants with mental disorders had consulted either religious or traditional healers before accessing psychiatric services. Despite the availability of biomedical practitioners and the socially subsidized medical care in Norway, some patients with mental disorders, especially the Sami populations still patronize traditional healers (10, 11).

A number of factors, including economic, demographic, social, cultural, psychological, clinical; have been associated with help seeking and care received by patients with mental disorders (12).

Studies in high income countries indicate that general practitioners and mental health professionals are central in pathways to psychiatric care whereas in Africa general duty doctors play a less prominent role but instead other (informal) health care providers, such as traditional healers, are more important in this regard (13).

Studies from Nigeria (14), Ethiopia (15), and South Africa have found significant delays in treatment in patients with psychiatric disorders, where traditional healers were the predominant first contact. In Western Nigeria, Lasebikan et al. (16), reported that 78.9% of the patients with mental disorders first utilized alternative sources of care (priests, spiritualists, natural therapists, or herbalists); 10% currently used such alternative sources in conjunction with formal mental health service; only 9.3% utilized general medical service as first contact, 7.8% utilized mental health service within general medical setting as first type of service while only 4.5% utilized specialist psychiatric service as first type of service. Abdulmalik and Sale (17) reported that in Northern Nigeria, close to 40% of children and adolescents seen in mental health service had contacted traditional and native healers. Among patients with schizophrenia in Lagos, Nigeria, Adeosun et al. (18) reported that the majority (69%) of the patients consulted spiritual or traditional healers as the first contact in the process of seeking care for the illness. At one time or the other during the course of the illness, 14.5% had sought help simultaneously from both traditional and spiritual healers, 42.8% from spiritual healers alone, and 11.6% from traditional healers alone. In the Niger Delta region of Nigeria, Jack-Ide et al. (19) reported that among patients with mental disorders who sought help at a local Psychiatric hospital, nearly half (48%) had first sought healing for mental health issues from religious leaders, primarily through prayers.

Of 238 patients who attended a mental health service in Ilorin, Nigeria, during a 1-month period, Abiodun (20) reported 40% had first contacted traditional or religious healers. The author suggests that use of psychiatric care in developing countries could be improved by training primary health care workers to give mental health education to the communities they serve. This suggestion seems out of tune with the findings of the study; traditional and faith healers are not formal primary health care workers. When patients develop mental disorders, they first visit traditional and faith healers, not formal primary care health workers.

In one study of the pathways of 159 patients with mental disorders to a tertiary psychiatric service in Nigeria, Acha and Odejide (21) reported that traditional and religious healers were consulted at some stage, mostly first contact, by many of the patients. Noting that patients who consulted traditional healers were more likely to arrive at a tertiary psychiatric service much later than those who consult other carers, the authors suggested that attempts to incorporate traditional medical care into the health care system must seek to improve their referral skill. It would seem that this suggestion jumps the gun. The traditional healers in that study had not been given any form of training in identifying and treating mental disorders before the issue of referral would become relevant.

Crawford and Lipsedge (22) reported that Zulu people in South Africa found western medicine useful for treating physical illness, but not mental illness principally because many mental health problems are considered to be understood only by traditional healers from their own culture. Africans in general are a very cultural and religious people who have great trust and confidence in their traditional and faith leaders.

In a study of help seeking by patients with a major depressive disorder in a South African Community, Andersson et al. (23) reported that 13% of the participants made use of traditional healers and were satisfied with the services. Sorsdahl et al. (24) in a study of mental health service use in South Africa, incorporated in the WHM Survey, concluded that alternative practitioners, including traditional healers and religious advisors, play an important role in the delivery of mental health care in South Africa. They found that 9% of the patients with mental disorders had used native healers and 11% had consulted faith healers.

Studies from Arab countries have also shown that the majority of patients with mental disorders consult traditional healers (faith healers, diviners, and herbalists) before seeking any biomedical doctor’s help or western treatment (25, 26). Similarly, in Asian countries patients with mental disorders first consult native healers while general practitioners play a less important role. (27–33). In one Chinese study, Zhang et al. (34) reported that only 5.5% of their patients with mental disorders first made contact with mental health service. In one study in Central India, Lahariya et al. (35) reported that 68.5% of patients with mental disorders first consulted faith healers.

It seems evident that mental health services are offered in a variety of settings by different groups, individuals, organizations, and health systems across the world. In many countries of the world, traditional and faith healers are the front line providers of (initial) mental health services. They can only be ignored at the peril of patients who need mental health services. Can traditional and faith healers be trained in the mhGAP intervention?

## Using Traditional Healers to Offer mhGAP

Some countries have contextualized the WHO mhGAP Intervention Guide. The WHO and World Organization of Family Doctors (WONCA) have argued that integrating mental health services into primary care is the most viable way of closing the treatment gap and ensuring that people get the mental health care they need (36). This stand presupposes that most people with mental disorders attend primary care and may be detected at that level. However, this is far from reality. In many developing countries as we have seen above, the majority of people with mental health problems use the services of faith and traditional healers, either alone or a step in the pathway to consulting experts in mental health. Therefore any program that would bridge the gap between mental health needs and services must take faith and traditional healing into account in developing countries.

Involvement of faith and traditional healers in orthodox medical care is a very complex issue. Patel (37) has argued that “*the greatest obstacle to a collaboration has been the mutual suspicion between the two sectors and the concerns of the biomedical sector and the religious establishment regarding the ‘unscientific’ and unorthodox practices of traditional healers*.” A major barrier in any such collaboration, according to Patel (37) includes the fact that there is a great diversity of traditional healers and moreover, there is lack of agreement on what constitutes evidence to guide policy and practice when the epistemologies of traditional medicine differ so vastly from that of biomedicine.

Although it is argued that some traditional healers do harm, orthodox western medical practitioners also do harm in various ways. The inescapable reality, Patel argues, is that traditional healers are far more numerous than biomedical providers and they play a particularly important role for mental health care. He concludes that an innovative system, guided by evidence and common sense, needs to be worked out where the collaboration between native healers and western orthodox medical practitioners will lead to complimentary rather than competitive therapy approaches.

According to Leavey and King (38), even though many clergy already provide pastoral care for emotionally distressed people, they may be reluctant to move further away from spiritual guidance – their “core business” – toward a more secular enterprise. They observed that the great heterogeneity of beliefs about suffering and healing among mainstream organizations, has particular resonance in relation to certain Evangelical and Pentecostal churches, which maintain deeply held beliefs and practices surrounding demonic possession, healing, and deliverance rituals. It is a great challenge for mental health professionals to engage with clergy who believe that sin or demonic possession lies at the root of a person’s illness. Leavey and King (38) conclude that it seems unlikely for clinical and legal reasons that services could or should collude with religious healing.

Robertson (39) argues that desirable as collaboration between western medical and traditional healers may be in Africa, there is a need to have expanded detailed studies of the knowledge, methods of practice of traditional healers, and how best to work out any collaboration.

In a qualitative study of traditional mental health care in Ghana, Ae-Ngibise et al. (40) observed that there are many reasons for the appeal of traditional and faith healers, including cultural perceptions of mental disorders, the psychosocial support afforded by such healers, as well as their availability, accessibility, and affordability. According to the authors, there are enormous barriers hindering collaboration between orthodox and traditional healers, including human rights and safety concerns, skepticism around the effectiveness of “conventional” treatments, traditional healer solidarity, and paradigmatic disjunctures and widespread skepticism between different treatment modalities. However, mutual respect and bi-directional conversations were the key ingredients for successful partnerships espoused by traditional healers in that study; therefore, promoting greater understanding, rather than maintaining indifferent distances may lead to more successful co-operation.

In a qualitative study of biomedical and traditional health practitioners in Cameroon, Wamba and Groleau (41) reported that biomedical practitioners and priests expressed reluctance in building reciprocal relationships with traditional healers and prophets. In a study of traditional and biomedical mental health practitioners in Tonga, Vaka et al. (42) reported that traditional healers had a negative view of the western-style system.

Some traditional healers lay claim to having cure for many diseases such as diabetes, hypertension retroviral disease, and epilepsy (43).

It is our contention that biomedical practitioners seem to put themselves on too high a pedestal. There is an eternal tendency for disdain against alternative medical practitioners. Yet we cannot wish away the fact that traditional healers have come to stay and are rendering unquantifiable mental health service. Until we have a total replacement alternative, we must have a way to accommodate traditional healers in the mental health service system in developing countries.

The claim of superiority over biomedical practitioners by some traditional healers creates the scenario of a supremacy battle.

Yet, strong and apparently reasonable as the arguments about the difficulty of collaboration may appear, it would seem that these difficulties have been unduly exaggerated. In reality, human nature is more universal than different, and suffering, distress, and how to remove them is a common thread across all people, wherever and however they are achieved. We are basically similar as human beings and the ultimate goal of every being is self actualizing. In whatever situation we look into, the basic principles of human relations are universal: empathic understanding, unconditional positive regard, and genuineness. It is not in doubt that many traditional and faith healers are bountifully imbued with these qualities. What needs to be done is to create a spirit of mutual understanding, respect, and co-operation.

The African Regional Strategy on promoting the role of traditional medicine in health systems strives to promote and encourage the collaboration between practitioners of traditional and conventional medicine (44, 45).

In a review of the participation of traditional healers in HIV/AIDS care in Africa, King and Homsy (46) reported encouraging results when these traditionalists are given some form of training and remain supported. Collaboration and partnerships between traditional and biomedical health professionals in HIV/AIDS care have been promising in many African countries including Botswana, Central African Republic, Guinea, Malawi, Mozambique, Tanzania, and Zambia (47). It has been amply shown that traditional health practitioners can be used to scale up HIV/AIDS care (48–51). Reports from Kenya, Mali, Senegal, South Africa, Uganda, and Zambia indicate that biomedical and traditional health practitioners can positively and successfully collaborate in the care of HIV/AIDS (45, 52).

The lessons already learned from HIV/AIDS collaboration could be extrapolated to mental disorders. In an East London project exploring traditional and faith based mental health healing by practitioners of African and Afro-Caribbean descent, it was found that adequate engagement could lead to a good collaboration with genuine faith healers accepting the treatment guide provided by biomedical healers (53). The project, Partnerships for Mental Health Development in Sub-Saharan Africa (PaM-D) (54), a hub aimed at creating an infrastructure to develop mental health research capacity in Sub-Saharan Africa works to integrate biomedical psychiatric care with traditional practice. With training and mutual respectful collaboration, the capacity of traditional healers to identify mental disorders and provide support, and referral to biomedical practitioners can be enhanced (2, 48, 55, 56).

The present WHO mhGAP is an opportunity to bring traditional and faith healers on board of the mental health service system in developing countries. With adapted training using the mhGAP intervention guide, it should be possible to get some traditional/faith healers to understand the core principles of some priority mental health problems identification, treatment, and referral. This is one sure way of bridging the treatment gap and closing the inequity and inequality chasm in mental health care.

## Conflict of Interest Statement

The authors declare that the research was conducted in the absence of any commercial or financial relationships that could be construed as a potential conflict of interest.

## References

[1] World Health Organization. The World Health Report 2001 – Mental Health: New Understanding, New Hope. Geneva: WHO (2001).

[2] Demyttenaere K Bruffaerts R Posada-Villa J Gasquet I Kovess V Lepine JP Prevalence, severity, and unmet need for treatment of mental disorders in the World Health Organization World Mental Health Surveys. JAMA (2004) 291:2581–90 10.1001/jama.291.21.2581 15173149

[3] Murray CJ Lopez AD Quantifying disability: data, methods and results. Bull World Health Organ (1994) 72:481–94 8062403 PMC2486704

[4] Whiteford HA Degenhardt L Rehm J Baxter AJ Ferrari AJ Erskine HE Global burden of disease attributable to mental and substance use disorders: findings from the Global Burden of Disease study 2010. Lancet (2013) 382:1575–1586 10.1016/S0140-6736(13)61611-6 23993280

[5] World Health Organization. Suicide Prevention, SUPRE, the WHO Worldwide Initiative for the Prevention of Suicide. Geneva: WHO (2009).

[6] Prince M Patel V Saxena S Maj M Maselko J Phillips MR No health without mental health. Lancet (2007) 370:859–77 10.1016/S0140-6736(07)61238-0 17804063

[7] WHO. Country Profile of Nigeria Mental Health Atlas of the WHO. Geneva: WHO (2011). p. 348–51

[8] World Health Organization. Mental Health Gap Action Programme: Scaling up Care for Mental, Neurological and Substance use Disorders. Geneva: WHO (2008). 26290926

[9] Gater R Jordanova V Maric N Alikaj V Bajs M Cavic T Pathways to psychiatric care in Eastern Europe. Br J Psychiatry (2005) 186:529–35 10.1192/bjp.186.6.529 15928365

[10] Kiil MA Salamonsen A Embodied health practices: the use of traditional healing and conventional medicine in a North Norwegian community. Acad J Interdiscip Stud (2014) 2:483–8 10.5901/ajis.2013.v2n3p483

[11] Sexton R Sorlie T Use of traditional healing among Sami psychiatric patients in the North of Norway. Int J Circumpolar Health (2008) 67:135–46 10.3402/ijch.v67i1.18250 18468265

[12] Gureje O Lasebikan VO Use of mental health services in a developing country. Soc Psychiatry Psychiatr Epidemiol (2006) 41:44–9 10.1007/s00127-005-0001-7 16341828

[13] Sorketti EA Zuraid NZ Habil MH Pathways to mental healthcare in high income and low-income countries. Int Psychiatry (2013) 10:45–9 PMC6735094 31507730

[14] Aghukwa CN Care seeking and beliefs about the cause of mental illness among Nigerian psychiatric patients and their families. Psychiatr Serv (2012) 63:616–8 10.1176/appi.ps.201000343 22638008

[15] Girma E Tesfaye M Patterns of treatment seeking behavior for mental illnesses in southwest Ethiopia: a hospital based study. BMC Psychiatry (2011) 11:138 10.1186/1471-244X-11-138 21859455 PMC3170592

[16] Lasebikan VO Owoaje ET Asuzu MC Social network as a determinant of pathway to mental health service utilization among psychotic patients in a Nigerian hospital. Ann Afr Med (2012) 11:12–20 10.4103/1596-3519.91010 22199042

[17] Abdulmalik IO Sale S Pathways to psychiatric care for children and adolescents at a tertiary facility in northern Nigeria. J Pub Health Afr (2012) 3:e4 10.4081/jphia.2012.e4 PMC5345448 28299078

[18] Adeosun I Adegbohun AA Adewumi TA Jeje OO The pathways to the first contact with mental health services among patients with schizophrenia in Lagos, Nigeria. Schizophr Res Treat (2013) 2013:769161 10.1155/2013/769161 24490072 PMC3893872

[19] Jack-Ide IO Makoro BBP Azibiri B Pathways to mental health care services in the Niger delta region of Nigeria. J Res Nurs Midwifery (2013) 2:22–9

[20] Abiodun OA Pathways to mental health care in Nigeria. Psychiatr Serv (1995) 46:823–6 7583485 10.1176/ps.46.8.823

[21] Acha RA Odejide OA Pathways to psychiatric care in Ibadan, Nigeria. Trop Geogr Med (1995) 47:125–9 7483003

[22] Crawford T Lipsedge M Seeking help for psychological distress: the interface of Zulu traditional healing and western biomedicine. Ment Health Relig Cult (2004) 7:131–48 10.1080/13674670310001602463

[23] Andersson LMC Schierenbeck I Strumpher J Krantz G Topper K Backman G Help-seeking behaviour, barriers to care and experiences of care among persons with depression in Eastern Cape, South Africa. J Affect Disord (2013) 151:439–48 10.1016/j.jad.2013.06.022 23890669

[24] Sorsdahl S Stein DJ Grimsrud A Seedat A Flisher AJ Williams DR Traditional healers in the treatment of common mental disorders in South Africa. J Nerv Ment Dis (2009) 197:434–41 10.1097/NMD.0b013e3181a61dbc 19525744 PMC3233225

[25] Sayed M Abosinaina B Rahim SIA Traditional healing of psychiatric patients in Saudi Arabia. Curr Psychiatr (1999) 6:11–23

[26] Salem MO Saleh B Yousef S Sabri S Help-seeking behaviour of patients attending the psychiatric service in a sample of United Arab Emirates population. Int J Soc Psychiatry (2009) 55:141 10.1177/0020764008093373 19240203

[27] Campion J Bhugra D Experiences of religious healing in psychiatric patients in South India. Soc Psychiatry Psychiatr Epidemiol (1997) 32:215–21 10.1007/BF00788241 9184467

[28] Giasuddin NA Chowdhury NF Hashimoto N Fujisawa D Waheed S Pathways to psychiatric care in Bangladesh. Soc Psychiatry Psychiatr Epidemiol (2012) 47:129–36 10.1007/s00127-010-0315-y 21076911

[29] Coton X Poly S Hoyois P Sophal C Dubois V The healthcare-seeking behaviour of schizophrenic patients in Cambodia. Int J Soc Psychiatry (2008) 54:328–37 10.1177/0020764008090286 18720893

[30] Phang CK Marhani M Salina A Prevalence and experience of contact with traditional healers among patients with first-episode psychosis in hospital Kuala Lumpur. Malays J Psychiatry (2010) 19(2):1–9

[31] Razali SM Najib MAM Help-seeking pathways among Malay psychiatric patients. Int J Soc Psychiatry (2000) 46:281–9 10.1177/002076400004600405 11201349

[32] Kurihara T Kato M Reverger R Tirta IG Pathway to psychiatric care in Bali. Psychiatry Clin Neurosci (2006) 60:204–10 10.1111/j.1440-1819.2006.01487.x 16594945

[33] Chong SA Abdin E Vaingankar JA Kwok KW Subramaniam M Where do people with mental disorders in Singapore go to for help? Ann Acad Med Singapore (2012) 41:154–60 22635279

[34] Zhang W Li X Lin Y Zhang X Qu Z Wang X Pathways to psychiatric care in urban north China: a general hospital based study. Int J Ment Health Syst (2013) 7:22 10.1186/1752-4458-7-22 24020825 PMC3852166

[35] Lahariya C Singhal S Gupta S Mishra A Pathways to psychiatric care in India. Indian J Psychiatry (2010) 52:333–8 10.4103/0019-5545.74308 21267367 PMC3025159

[36] World Health Organization. Integrating Mental Health into Primary Care – A Global Perspective. Singapore: WHO/WONCA (2008).

[37] Patel V Traditional healers for mental health care in Africa. Global Health Action (2011) 4:7956 10.3402/gha.v4i0.7956 PMC3150105 21845145

[38] Leavey G King M The devil is in the detail: partnerships between psychiatry and faith-based organizations. Br J Psychiatry (2007) 191:97–8 10.1192/bjp.bp.106.034686 17666491

[39] Robertson BA Does the evidence support collaboration between psychiatry and traditional healers? Findings from three South African studies. S Afr Rev (2006) 9:87–90 10.4314/ajpsy.v9i2.30210

[40] Ae-Ngibise K Cooper S Adiibokah E Akpalu B Lund C Doku V The MhaPP Research Programme Consortium. Whether you like it or not people with mental problems are going to go to them: a qualitative exploration into the widespread use of traditional and faith healers in the provision of mental health care in Ghana. Int Rev Psychiatry (2010) 22:558–67 10.3109/09540261.2010.536149 21226644

[41] Wamba A Groleau D Constructing collaborative processes between traditional, religious, and biomedical health practitioners in Cameroon Nordic. J Afr Stud (2012) 21:49–74

[42] Vaka S Stewart MW Foliaki S Tu’itahi M Walking apart but towards the same goal? The view and practices of Tongan traditional healers and western-trained Tongan mental health staff. Pac Health Dialog (2009) 15:89–95 19585738

[43] Edward S John C Health and wellness in Southern Africa: incorporating indigenous and western healing practices. Int J Psychol Couns (2012) 4:59–67

[44] World Health Organization. Promoting the Role of Traditional Medicine in Health Systems: A Strategy for the African Region. WHO Regional Office for Africa, Temporary location, Harare, Zimbabwe. (Document AFR/RC50/9 and Resolution AFR/RC50/R3). Geneva: WHO (2001).

[45] Busia K Kasilo OMJ Collaboration between traditional health practitioners and conventional health practitioners: some country experiences. Afr Health Monit (2010) (13).

[46] King R Homsy J Involving traditional healers in AIDS education and counseling in sub-Saharan Africa – a review. AIDS (1997) 11:S217–25 9451988

[47] King R Collaboration with Traditional Healers in HIV/AIDS Prevention and Care in Sub-Saharan Africa – A Literature Review. Geneva: UNAIDS (2000).

[48] Kaboru BB Falkenberg T Ndubani P Höjer B Vongo R Brugha R Can biomedical and traditional health care providers work together? Zambian practitioners’ experiences and attitudes towards collaboration in relation to STIs and HIV/AIDS care: a cross-sectional study. Hum Resour Health (2006) 4:16 10.1186/1478-4491-4-16 16846497 PMC1540435

[49] Bodeker G Kabatesi D King R Homsy J Regional task force on traditional medicine and AIDS. Lancet (2000) 355:1284 10.1016/S0140-6736(05)74722-X 10770339

[50] Homsy J King R Balaba D Kabatesi D Traditional health practitioners are key to scaling up comprehensive care for HIV/AIDS in sub-Saharan Africa. AIDS (2004) 18:1723–5 10.1097/01.aids.0000131380.30479.16 15280784

[51] UNAIDS. Collaboration with Traditional Healers in HIV/AIDS Prevention and Care in Sub-Saharan Africa: A Literature Review. In UNAIDS Best Practice Collection. Geneva: Joint United Nations Programme on AIDS (2000).

[52] Chirwa BU Sivile E Enlisting the support of traditional healers in an AIDS education campaign in Zambia. Int Q Community Health Educ (1989) 9:221–9 10.2190/DEQP-8A5W-ALU0-4XV4 20841206

[53] Hills D Aram E Hinds D Warrington C Brissett L Stock L Traditional Healers Research Project. Final Report of the Tavistock Institute of Human Relations. London: The Tavistock Institute (2013).

[54] Partnerships for Mental Health Development in Sub-Saharan Africa (PaM-D). Available from: http://www.pam-d.org/

[55] Chadda RK Agarwal V Singh MC Raheja D Help seeking behaviour of psychiatric patients before seeking care at a mental hospital. Int J Soc Psychiatry (2000) 47:71–8 10.1177/002076400104700406 11694059

[56] Mkize LP Cur M Uys LR Pathways to mental health care in KwaZulu-Natal. Curationis (2004) 27:62–71 10.4102/curationis.v27i3.1001 15777031

